# Molecular Epidemiology of HIV-1 and HTLV-1/2 Among Female Sex Workers in Four Cities in the State of Para, Northern Brazil

**DOI:** 10.3389/fmicb.2020.602664

**Published:** 2020-11-11

**Authors:** Ronaldo Lopes de Souza, Marcelo Victor Serejo Pereira, Rachel Macedo da Silva, João Bráullio de Luna Sales, Danilo Cesar Lima Gardunho, Jacqueline Cortinhas Monteiro, Leonardo Quintão Siravenha, Anderson Luiz Bessa da Luz, Ricardo Roberto de Souza Fonseca, Aldemir Branco Oliveira-Filho, Marluísa de Oliveira Guimarães Ishak, Ricardo Ishak, Luiz Fernando Almeida Machado

**Affiliations:** ^1^Campus de Abaetetuba, Universidade Federal do Pará, Abaetetuba, Brazil; ^2^Programa de Pós-Graduação em Biologia de Agentes Infecciosos e Parasitários, Instituto de Ciências Biológicas, Universidade Federal do Pará, Belém, Brazil; ^3^Laboratório de Virologia, Instituto de Ciências Biológicas, Universidade Federal do Pará, Belém, Brazil; ^4^Instituto de Ciências Exatas e Naturais, Universidade Federal do Pará, Belém, Brazil; ^5^Grupo de Estudo e Pesquisa em Populações Vulneráveis, Instituto de Estudos Costeiros, Universidade Federal do Pará, Bragança, Brazil

**Keywords:** HIV, HTLV, epidemiology, female sex workers, Brazil

## Abstract

Female sex workers (FSWs) represent a key population for the acquisition of sexually transmitted infections (STI) due to their social vulnerability and the risks associated with their occupation. This study was conducted to describe the sociodemographic characteristics and sexual behavior among FSWs in cities in northern Brazil, to determine the prevalence of human immunodeficiency virus 1 (HIV-1) and human T-cell lymphotropic virus (HTLV-1/2) infections and to identify the circulating subtypes of these agents in this key population. A cross-sectional study using the Time Location Sampling (TLS) method was conducted among 339 FSWs in cities in the state of Pará from 2005 to 2006. Serological and molecular tests were performed to identify infections and viral subtypes, and bivariate and multivariate analyses were conducted to identify risk factors. Most FSWs were young, single, less educated and had at least one child. The prevalence of antibodies against HIV-1 and HTLV-1 was 2.3 and 1.7%, respectively. HIV-1 subtypes B (87.5%) and F1 (12.5%) were identified among FSWs, as were Cosmopolitan subtype (1a) and Transcontinental subgroup (A). Unprotected sex and illicit drug use were associated with HIV-1 and HTLV-1 infections using bivariate and multivariate analyses, and age ≥27 years was associated only with HIV. The important information highlighted here clearly indicates that the lack of actions to control and prevent pathogens in FSWs and the lack of strategies for health promotion in key populations can further aggravate the epidemiological scenario of viral infections in remote areas with low human development indices. Neglecting these facts may be causing the spread of these two viruses and their respective subtypes in the general population of northern Brazil.

## Introduction

In South America, Brazil is highlighted for having the largest territorial extension, the largest population and immense economic importance. Another important feature is the wide gap of socioeconomic inequality among Brazilians (Campello et al., 2018). The combination of large territorial area and socioeconomic inequality creates a big challenge for governments to provide social inclusion, access to appropriate education and healthcare (Campello et al., 2018; de Souza et al., 2020). An example of this challenge has been reports of the absence or precarious implementation of policies and strategies for the control and prevention of infectious agents among vulnerable population groups in Brazil, especially in remote areas (Oliveira-Filho et al., 2019a, 2020).

Female sex workers (FSWs) represent a key population for the acquisition of sexually transmitted infections (STIs) due to their social vulnerability and the risks associated with their occupation, including multiple sexual partners, inconsistent use of condoms, use of injecting and non-injecting drugs and co-infections with other STIs (World Health Organization [WHO], 2016; Cavalcante et al., 2019; da Costa et al., 2019; Frade et al., 2019a). The role of FSWs is of relevance as they usually spread infectious agents, including Human immunodeficiency virus 1 (HIV-1) and human T-cell lymphotropic virus (HTLV), from high-risk groups to the general population (Malta et al., 2010; Oliveira-Filho et al., 2019b, 2020). The monitoring of infectious agents in populations with a potential to transmit them, including STIs by FSWs, is of paramount importance to establish control and prevention measures and to direct specific actions for health promotion (Cavalcante et al., 2019; Frade et al., 2019b; Oliveira-Filho et al., 2019a). In the Pará state, the prevalence of HIV and HTLV infection was 0.3 and 0.6% respectively in pregnant adolescents (Guerra et al., 2018) and the prevalence of HIV in the general population of in Marajoì Island was 0.6% (Vallinoto et al., 2016). In a study carried out in the general population of the city of Belém, capital of the state of Pará, the prevalence of HTLV-1 and HTLV-2 infection was 1.4 and 0.5% respectively (Silva et al., 2018).

The average world prevalence of HIV-1 among FSWs is close to 12%, and approximately 15% of people who have unprotected sex with FSWs are infected with HIV-1 (Baral et al., 2012). In Brazil, HIV-1 is found among 5% (Damacena et al., 2011) of FSWs, with a prevalence range of 1.0–16.1% according to the geographic region in the country (Malta et al., 2010; Schuelter-Trevisol et al., 2013; Fernandes et al., 2014; Ferreira-Juìnior et al., 2018; da Costa et al., 2019). The majority of the prevalence investigations with FSWs are from the South, Southeast and Central Brazil (Malta et al., 2010; Schuelter-Trevisol et al., 2013; Fernandes et al., 2014; Ferreira-Juìnior et al., 2018). Recently, a high prevalence of HIV-1 (16.1%) among FSWs in northern Brazil was described and associated with risky sexual behavior, use of illicit drugs and socioeconomic characteristics (da Costa et al., 2019). HIV-1 subtypes B and F1 and BF1 recombinant were detected, as well as a high prevalence of transmitted drug resistant mutations (47.6%) (da Costa et al., 2020). Although HTLV-1/2 infections are endemic in the State of Para and associated with risky sexual behavior (Oliveira-Filho et al., 2019a; Ishak et al., 2020), to date, there have been no epidemiological investigations of the virus among FSWs.

The present investigation was conducted to describe sociodemographic characteristics, sexual behavior, and the prevalence and circulating molecular subtypes of HIV-1 and HTLV-1/2 among FSWs of four cities in the State of Para.

## Materials and Methods

### Study Population

**FIGURE 1:**
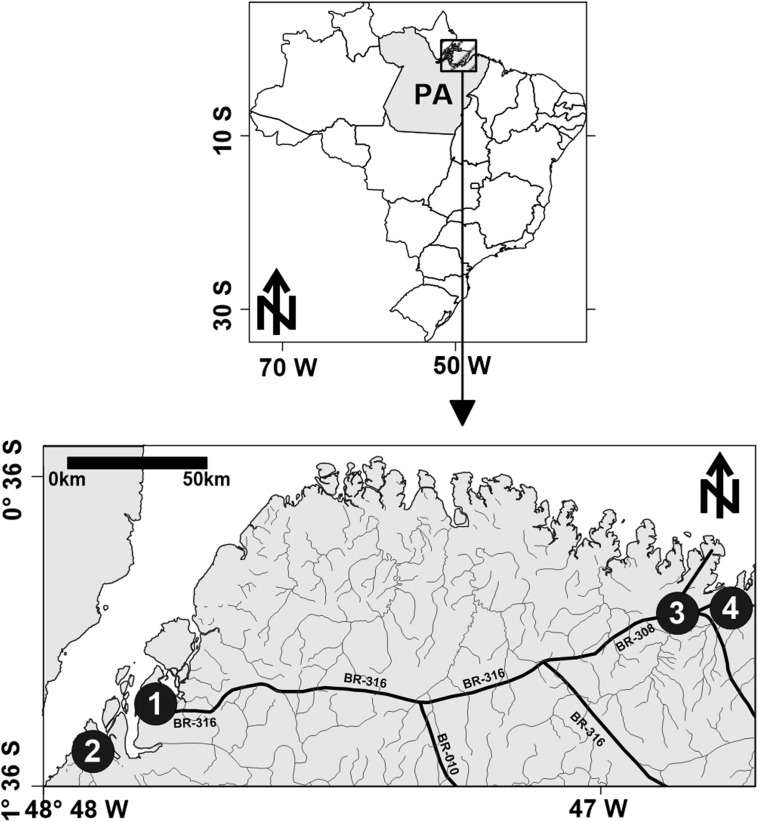
Geographic location of cities in the state of Pará (PA), northern Brazil. The numbers indicate the cities of Belém (1), Barcarena (2), Bragança (3), and Augusto Correa (4).

FSWs (*n* = 339) residing in the State of Para, northern Brazil in the municipalities of Belem (*n* = 105), Barcarena (*n* = 105), Bragança (*n* = 98) and Augusto Correa (*n* = 31) were invited and agreed to participate in the present investigation during the period of April 2005 to December 2006. The cities are located in the most densely populated areas in the North region as a consequence of the historical process of occupation and development of the region (Figure 1).

### Study Design

Belém is the capital of the state of Pará (1,552,275 inhabitants) and, together with Barcarena (121,190 inhabitants), is located in the metropolitan region of Belém. Bragança (125,184 inhabitants) is the main city in the Northeast area of the State together with Augusto Correa (45,435 inhabitants).

The investigation was a descriptive cross-sectional epidemiological study in which an extensive mapping of working places, sex trade venues (STV), in the cities of Belém (*n* = 18), Barcarena (*n* = 10), Bragança (*n* = 8) and Augusto Correa (*n* = 4) were plotted, and the FSWs were accessed using Time Location Sampling (TLS) (Semaan et al., 2002).

Observation of these sites and reports from residents and clients provided information related to the number of FSWs at STV, days and times of increased client flow during the week, and customs and possible difficulties of access to FSWs. After the location of STVs the initial approach of the FSWs was performed during 2 days along the week, always at dusk, before the peak of client movement. Initially, a quick lecture about the most important STIs and prevention and control methods was performed and following that FSWs were informed about the study. Those who accepted to participate signed a consent form, and blood was collected. Demographic data (age, schooling, marital status and number of children) as well as information on sexual behavior (sexual practices, condom use, number of sexual partners per week) and drug use (illicit and licit) were provided by filling out a pre-tested standardized semi-structured questionnaire. The inclusion criteria were: sexually active women, sexual programs in exchange for money, and consent to participate (signature of the informed consent form). The exclusion criteria were: women who were in the place of destination but were not sex workers and male and transgender sex worker.

### Serological Assays

A blood sample (5 mL) was collected in a vacuum system tube with EDTA as an anticoagulant and was transported to and processed in the Laboratory of Virology of the Institute of Biological Sciences of UFPA (Belém, Pará, Brazil). After blood centrifugation for 5 min at 5,000 rpm, plasma and leucocyte samples were isolated and stored at −20°C prior to use.

Plasma was tested for the presence of antibodies against HIV-1 (HIV-1/2 Ab-Capture ELISA Test System, Ortho Diagnostic Systems Inc., United States) and HTLV-1/2 (HTLV-1/2 Ab-Capture ELISA Test System, Ortho Diagnostic Systems Inc., United States) using an enzyme-linked immunosorbent assay (ELISA), according to the manufacturer’s protocol. HIV-1 infection was confirmed by Western blot (Western blot MP Diagnostics, United States), and HTLV-1 and HTLV-2 infections were confirmed using a nested polymerase chain reaction (PCR).

### Molecular Assays

Positive samples for antibodies against HIV-1 were selected for the isolation of nucleic acid using a Puregene kit (Puregene, Gentra Systems, Inc., United States) according to the manufacturer’s instructions. DNA was used as the target for a nested PCR for amplification of 297 bp of the protease gene (codons 1-99). The primers used were DP10/DP11 (first round) and DP16/DP17 (second round) as previously described (Janini et al., 1996). The first-round PCR used 400 ng of extracted DNA, 125 mM of each dNTP (Invitrogen^TM^, United States), 5 pmol/μl of each of the two external primers, 2.5 mM MgCl_2_, and 10 μl PCR buffer (Invitrogen^TM^, United States) in a final volume of 50 μl. Reactions were carried out in a thermocycler (Eppendorf Scientific, Inc., Westbury, NY, United States) for 5 min at 94°C, followed by 35 cycles at 94°C (40 sec), 50°C (40 sec), 72°C (1 min), and extension for 10 min at 72°C. Five microliters of amplified product was used in the nested PCR using a set of internal primers in a final volume of 50 μl and using the same temperature and incubation periods that were employed in the first reaction.

Positive samples for antibodies against HTLV-1/2 were tested using a nested PCR to amplify the 5′ LTR region. The 1st and 2nd round PCR were run in a final volume of 50 μL containing 400 ng of extracted DNA, 10 μM of each dNTP, 20 pmol/μL of each primer, 50 μM MgCl_2_, 1× buffer (50 mM KCl, 10 mM Tris-HCl pH 8.3) and 5 U of Taq DNA polymerase. In each amplification reaction, after initial denaturation at 94°C for 5 min, 35 cycles were performed, with 40 s at 94°C, followed by 30 s at 57°C and 1 min at 72°C. These 35 cycles were followed by a final extension for 15 min at 72°C. The primers used were LTR-I.01/LTR-I.02 (first round) and LTR-I.03/LTR-I.04 (second round) for HTLV-1 and F-IILTR/Long Gag (first round) and Mo15/MSW-Gag (second round) for HTLV-2 (Laurentino et al., 2005). DNA was subjected to a Restriction Fragment Length Polymorphism (RFLP) targeting 159 bp of HTLV *pX* region to discriminate infection by HTLV-1 and HTLV-2, as previously described (Laurentino et al., 2005).

The PCR products (HIV-1 and HTLV-1/2) were electrophoresed on a 1.5% agarose gel (200 V, 120 Amp, and 45 min) and purified by the QIA Quick Purification Kit (Qiagen Inc., Germantown, MD, United States) prior to direct sequencing of the product. Each sample was sequenced twice in both directions using the ABI Prism BigDye^TM^ Terminator Ready Reaction Cycle Sequencing kit, version 3.1 (Applied Biosystems, Foster City, CA, United States) and ABI 3130 automated sequencer (Applied Biosystems, Foster City, CA, United States), following the manufacturer’s protocol. The sequences obtained in this study were deposited in GenBank (MN736950 - MN736953 and MT651208 - MT651215).

### Identification of Viral Subtypes

Nucleotide sequences obtained from FSWs with HIV-1 were aligned and edited using the BioEdit and AliView programs. The sequences originating from the protease gene of HIV-1 and the 5′ LTR region of HTLV-1/2 were used, together with other strains described in the GenBank, to establish the phylogenetic relationship. Maximum-likelihood (ML) phylogenetic trees were reconstructed with PhyML 3.1 (Guindon et al., 2010) under the best nucleotide substitution model and selected by the SMS (Smart Model Selection) software integrated into the PhyML Web server in order to verify the clustering of HIV-1 and HTLV-1/2 sequences. The heuristic tree search was performed using the SPR branch-swapping algorithm, and the branch support was calculated with the approximate likelihood-ratio (aLRT) SH-like test. Phylogenetic trees were drawn with FigTree 1.4.4.^1^ HIV-1 subtypes were also determined using REGA (de Oliveira et al., 2005) and COMET (Struck et al., 2014). The presence of recombination in HIV-1 sequences was performed by bootscanning analysis (sliding window of 250 bp, incremental steps of 10 bases, and the Kimura two-parameter model using Simplot 3.5.1 software (Lole et al., 1999). Bootstrap support was calculated based on 100 re-samplings.

### Statistical Analysis

The study data collected were entered into an Excel database and converted to SPSS. Confidence intervals were computed to estimate the prevalence of HIV-1 and HTLV-1/2 infection rates. Odds ratios (OR) and associated 95% confidence intervals (CI) were used as measures of the strength of the independent association between HIV + (outcome) and HTLV-1/2 + (outcome) and individual variables and the variation of this strength. Both bivariate and multivariate logistic regression models were used to assess the association of the independent variables with outcomes. Variables associated with the outcome, with *p*-values <0.20 using bivariate analysis, were entered into a backward stepwise logistic regression model (multivariate analysis) (Oliveira-Filho et al., 2019a). The fit of the final model was assessed using the Hosmer-Lemeshow goodness-of-fit test. Statistical significance was achieved when *p*-value (*p*) <0.05. Statistical analyses and procedures were performed using SPSS 20.0 for Windows.

### Ethical Aspects

All procedures were performed in accordance with the guidelines and regulatory standards for research involving human subjects of the National Health Council. This study was approved by the Ethics Committee on Human Research of the Fundação Centro de Hemoterapia e Hematologia do Pará (Protocol Number 01/2005).

## Results

The demographic and epidemiological characteristics of the 339 persons involved in the investigation are shown in Table 1. The mean age of the FSWs was 27.2 years old (range from 15 to 71). From this mean age value (total), a limit (≥27 years) was established to assess the association of this variable with viral infections. In addition, most FSWs were single (84.1%), mothers (68.1%; mean of 2.4 children), with less than 8 years of school years, earned less than a minimum wage (52.2%), did not use illicit drugs (87.0%), reported the use of condoms (59.0%) and had a mean of 9.6 clients per week. Regarding the use of illicit drugs, the use of non-injectable drugs was reported by some FSWs (13.0%).

**TABLE 1:** Characteristics of the population sample of FSWs in four cities in the state of Pará, northern Brazil (2005–2006).

Characteristics	Cities								Total (*n* = 339)	
	Bragança (*n* = 98)		Augusto corrêa (*n* = 31)		Belém (*n* = 105)		Barcarena (*n* = 105)			
	*N*	%	*N*	%	*N*	%	*n*	%	*N*	%
**Age (years)**										
Average	24.7		24.9		31.7		27.4		27.2	
Range	15–54		16–56		15–71		16–51		15–71	
**Marital status**										
Single	87	89.8	20	64.5	83	79.1	95	90.5	285	84.1
Married	11	10.2	11	35.5	22	20.9	10	9.5	54	15.9
**Children**										
Yes	57	58.4	20	64.5	98	93.4	56	53.5	231	68.1
No	41	41.6	11	35.5	7	6.6	49	46.5	108	31.9
**Number of children**										
Average	2.4		2.5		2.8		2.8		2.6	
**Schooling (years)**										
≤8	90	91.8	29	93.5	86	81.9	74	70.4	279	82.3
>8	8	8.2	2	6.5	19	18.1	31	29.6	60	17.7
**Monthly income**										
Up to 1 wage*	41	41.8	23	74.2	43	40.9	38	36.2	145	42.8
More than 1 wage	57	58.2	8	25.8	62	59.1	67	63.8	194	52.2
Use of illicit drugs**										
Yes	0	0	0	0	25	23.8	19	18.1	44	13.0
No	98	100.0	31	100.0	80	76.2	86	81.9	295	87.0
Condom use***										
Yes	60	61.2	16	51.6	72	68.5	52	49.5	200	59.0
No	38	38.8	15	48.4	33	31.5	53	50.5	139	41.0
**Clients per week**										
Average	12.0		8.9		10.5		7.0		9.6	
Range	11–42		8–23		15–50		7–20		7–50	
**HIV infection**										
Positive	0	0.0	0	0.0	7	6.6	1	0.9	8	2.4
Negative	98	100.0	31	100.0	98	93.4	104	99.1	331	97.6
**HTLV infection**										
Positive	0	0.0	1	0.9	4	3.8	1	0.9	6	1.8
Negative	98	100.0	30	99.1	101	96.2	104	99.1	333	98.2

*Average of the Brazilian minimum wage from 2005 to 2006 = R$ 325,00 (equivalent to US$ 81,00). **Only the use of non-injectable drugs was reported. ***Last 30 days.

**FIGURE 2:**
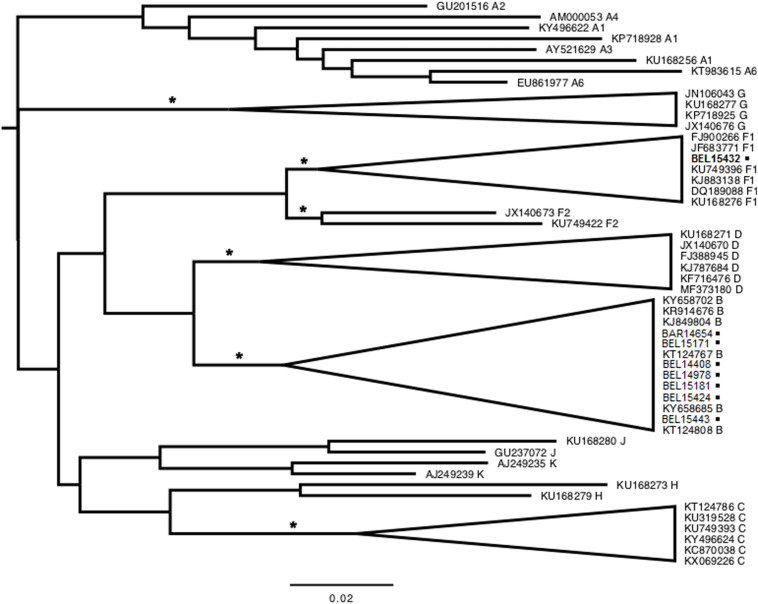
Phylogenetic tree constructed by maximum likelihood using PR sequences (297 base pairs) belonging to the HIV-1 protease region detected in female sex workers in cities of the state of Pará, northern Brazil. The tree was rooted at the midpoint. Asterisks point to key nodes with high support (aLRT ≥ 0.90). Samples from this study can be identified by city acronym [Belém (BEL) and Barcarena (BAR)] + number, and highlighted with black squares.

**FIGURE 3:**
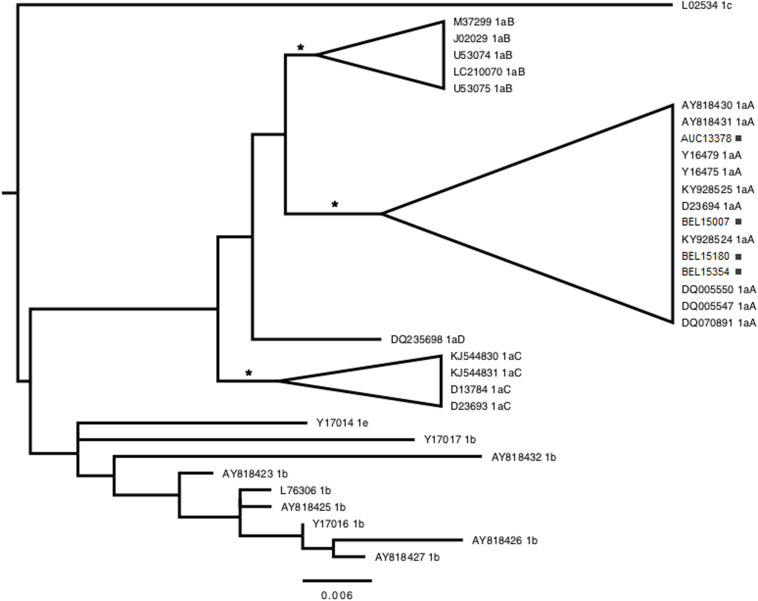
Phylogenetic tree constructed by maximum likelihood using long terminal repeat (LTR) sequences (548 base pairs) belonging to the HTLV-1 detected in female sex workers in cities of the state of Pará, northern Brazil. The tree was rooted at the midpoint. Asterisks point to key nodes with high support (aLRT ≥ 0.90). Samples from this study can be identified by city acronym [Augusto Correa (AUC), Barcarena (BAR), or Belém (BEL)] + number, and highlighted with black squares.

Eight (2.4%, 95% CI: 0.0–6.7) FSWs tested positive for anti-HIV-1/2 antibodies, and six (1.8%, 95% CI: 0.0–5.8) tested positive for anti-HTLV-1/2 antibodies, with the results confirmed using Wb and PCR assays, respectively. One HIV-1-infected woman worked in Barcarena and the other seven in Belém (Table 1). Subtyping was confirmed by the different methods used, and seven samples (87.5%) were from subtype B and one subtype F1 (12.5%) (Figure 2). No HIV-1 recombinants were identified. By contrast, one HTLV-infected woman worked in Augusto Correa, one in Barcarena and four in Belem (Table 1). Four samples were amplified and were typed as HTLV-1, Cosmopolitan subtype 1 (HTLV-1a) and Transcontinental subgroup A (Figure 3), however, in two HTLV positive samples, the amplification of 5′LTR region was not successful. Neither HTLV-2 infection nor HIV-1/HTLV-1 coinfection were detected. Furthermore, unprotected sex and illicit drug use were associated with HTLV-1 using bivariate and multivariate analyses. Regarding HIV-1, three factors were identified by bivariate and multivariate analysis: age ≥27 years, unprotected sex and illicit drug use (Table 2). The Hosmer-Lemeshow test indicated that these models of factors associated with HIV-1 (_HL_χ^2^ = 1.9; *p*-value = 0.4) and HTLV-1 (_HL_χ^2^ = 0.8; *p*-value = 0.4) had good overall fits. Factors not associated with HIV and HTLV infections can be accessed from additional files (Supplementary Tables 1 and 2).

**TABLE 2:** Bivariate and multivariate analysis of factors associated with HIV and HTLV among female sex workers in the state of Pará, northern Brazil (2005–2006).

Characteristics	*N*	HIV + (%)	HIV (%)	Bivariate analysis		Multivariate analysis		_HL_χ^2^ (*p*-value)
				*p*-value	OR (95% CI)	*p*-value	aOR (95% CI)	
Total	339	8 (2.4)	331 (97.6)					
Age ≥ 27 years	59	5 (8.5)	54 (91.5)	0.02	8.5 (2.0–33.6)	0.01	7.1 (2.6–18.3)	1.9 (0.4)
Use of illicit drugs	44	6 (13.6)	38 (86.4)	<0.01	23.2 (4.5–68.3)	<0.01	18.8 (13.4–28.7)	
Unprotected sexual relations^+^	121	7 (5.8)	116 (94.2)	<0.01	13.1 (1.7–59.5)	<0.01	13.2 (9.6–21.5)	

**Characteristics**	***N***	**HTLV + (%)**	**HTLV–(%)**	***p*-value**	**OR (95% CI)**	***p*-value**	aOR** (95% CI)	**_HL_χ^2^ (*p*-value)**

Total	339	6 (1.8)	333 (98.2)					
Use of illicit drugs	44	3 (6.8)	336 (93.2)	0.03	7.1 (1.4–34.2)	0.02	7.6 (2.5–12.4)	0.8 (0.4)
Unprotected sexual relations^+^	121	5 (4.1)	334 (95.9)	0.02	9.5 (1.1–61.1)	0.01	9.3 (4.9–14.2)	

*^+^Last 30 days. _HL_χ^2^: Hosmer-Lemeshow Test. 95% CI, 95% confidence intervals; OR, odds ratio; aOR, adjusted OR.*

## Discussion

The metropolitan area of Belém and the cities of the northeast of the State have been the main areas of commerce and development in Para since the foundation of the city in 1616. In the cities investigated, there have been reports of sex trade since the 18th century as a result of the intense flow of people and products, particularly in the vicinity of the main ports of entry and exit (by horses, boats, and later with trains and roads). In spite of the importance of the population group, little information is available on FSWs in the North of Brazil and their role in the dissemination of HIV-1 and HTLV-1/2. Relevant information was collected in the present study as evidence of the absence of effective actions to diagnose and take prevention and control measures against sexually transmitted infections. The absence of strategies for health promotion among key population groups certainly aggravates the epidemiological scenario of these two viral infections in remote areas with low human development indices.

In this study, we observed that most FSWs were young, single, had children and had few years of schooling similar to that observed in several Brazilian localities, such as Goiânia (Caetano et al., 2013), Campo Grande (Ferreira-Juìnior et al., 2018) and cities of Santa Catarina (Schuelter-Trevisol et al., 2007, 2013), which shows a uniform demographic profile among the various regions of Brazil. One of the differences in relation to the number of school years was observed in Goiânia, where FSWs reported at least 11 years of schooling (Caetano et al., 2013).

The majority of participants in this study did not report using illicit or injecting drugs. It is important to highlight the change in the epidemiological behavior of the community in one decade. A significant change occurred from 2005–2006 compared to 2016–2018, when the use of illicit drugs as a risky behavior was associated with the spread of HIV-1 and other infectious agents among FSWs in northern Brazil, such as hepatitis B virus (HBV), hepatitis C virus (HCV) and *Treponema pallidum* (Cavalcante et al., 2019; da Costa et al., 2019; Frade et al., 2019a; Oliveira-Filho et al., 2019a).

Most FSWs reported having a large number of partners and making use of condoms in sexual relations at the same frequency as those reported in other regions of Brazil (Caetano et al., 2013; de Matos et al., 2013; Fernandes et al., 2014). The social and economic vulnerability of FSWs in northern Brazil indicates their need to maintain a large number of sexual partners for obvious financial reasons, but the risk is somewhat increased by unprotected sex, which is sometimes demanded by clients in exchange for more money, although it increases the risk of acquiring and spreading infectious agents, including HIV-1, HBV and HCV (Cavalcante et al., 2019; da Costa et al., 2019; Frade et al., 2019b; Oliveira-Filho et al., 2019a).

The general prevalence of HIV-1 infection detected was high (2.3%) when compared to the figures reported for the general population in Brazil (0.6%) (Ministério da Saúde Brasil, 2018) but was within the range for Brazilian FSWs (1.0–16.1%) (Malta et al., 2010; Damacena et al., 2011; Schuelter-Trevisol et al., 2013; Fernandes et al., 2014; da Costa et al., 2019) and those from South American countries (0.0–4.5%) (Bautista et al., 2006, 2008, 2009). Unfortunately, the figures were not maintained over the years; more recently, a high prevalence of HIV-1 was described among FSWs in the city of Bragança (16.1%) (da Costa et al., 2019) in a follow up surveillance program from our laboratory. There are several points that can be related to this difference from 0% (present study) to 16.1% (da Costa et al., 2019). Initially, the information revealed by this study was obtained from 2005 to 2006. On the other hand, da Costa et al. (2019) obtained information on HIV-1 among FSWs in 2017. Second, the sampling methods used to access FSWs in the studies were different: Time Location Sampling (present study, *n* = 98) and Respondent-Driven Sampling (da Costa et al., 2019, *n* = 137). Third, in this period of approximately 10 years, the city of Bragança has undergone changes in the economic area. In addition to the increase in the number of port areas (consequently, an increase in sex trade points), another important change was the intensification of tourism in the municipality (based on picturesque scenery, beaches, rivers, and islands). These events have enabled an increase in the sex trade in the city of Bragança and, considering the socio-economic conditions of FSWs, the occurrence of unprotected sex and the use of illicit drugs has increased and likely has been responsible for the expansion of HIV-1. Despite different sampling methods between the two studies, other events may be strongly associated with the change in the prevalence of HIV infections among FSWs in the city of Bragança. In the study by da Costa et al. (2019), there is a report of the offer of sexual services through apps and social networks, and waived condom use if the client pays more for the sexual encounter. The findings of the present study are very important to indicate: (1) the epidemiological changes occurring in the region, and (2) the lack of effective care and interventions that could have blocked the entry and spread of the virus in the community. In summary, this stresses the importance of continuous prevalence studies, which have shown a disproportionate and unprecedent expansion of the HIV-1 epidemic from the capital to the interior of the state of Pará, as seen in Bragança. Brazil guarantees free antiretroviral treatment to all people living with HIV-1/AIDS since 2013, independently of their viral load, accordingly to the “test and treat” approach. Thus, the early diagnosis of HIV-1 infection in highly vulnerable population, and the administration of antiretroviral treatment, are important tools to decrease HIV-1 transmission.

Finding HIV-1 subtypes B and F1 among FSWs is compatible with previous reports in populations in the states of Amazonas, Amapá and Pará, northern Brazil (Machado et al., 2009; Cunha et al., 2012). However, the data also show an expansion in the variability of HIV-1 molecular subtypes with the recent detection of subtypes B (predominant), F1, and recombinant BF1 among FSWs in the city of Bragança, and a high rate of drug resistance mutations (da Costa et al., 2020). This is the clearest evidence that HIV is spreading in the interior of the state of Pará and undergoing recombination and accumulating mutations through unprotected sex.

The investigation of HTLV-1/2 infections among FSWs is the first approach in Amazon Region of Brazil, particularly in a highly endemic area of HTLV infection (Ishak et al., 2020). The present study observed a higher general seroprevalence of HTLV-1 than those reported in the population of men who have sex with men (0.7%) (Malta et al., 2010); candidates for blood donation in the Northeast of Brazil (1.2%) (Ribeiro et al., 2018), the Brazilian state of Amazonas (0.13%) (Morais et al., 2017) and in non-FSW women who had a similar socioeconomic profile, such as pregnant teenagers (0.6%) (Guerra et al., 2018) and women from Belém, Pará (1.4%) (Silva et al., 2018) and parturients (0.4%) of Salvador, Bahia (Vargas et al., 2020). However, the seroprevalence was much lower than the figure recorded among illicit drug users in the state of Pará (6.4%) (Oliveira-Filho et al., 2019b). It is possible that such a figure is a consequence of the higher level of risky behavior, such as injecting drug use and multiple sexual partners. Co-infections with the two viruses were not observed, unlike previous investigations with HIV-1-infected persons in the states of Para and Amapa, in which HTLV-1 and HTLV-2 co-infections were detected (Vallinoto et al., 1998; Laurentino et al., 2005; Ishak et al., 2020). This indicates a low prevalence of co-infections among FSWs in the North region of Brazil.

HTLV-1, but not HTLV-2, was identified among FSWs in three cities of the state of Pará, suggesting greater spread of the virus, although the prevalence was lower than HIV-1. Cosmopolitan subtype, Transcontinental subgroup, is the major subtype in circulation in the State of Para (Laurentino et al., 2005; de Aguiar et al., 2017; Ishak et al., 2020) and in other Brazilian regions (Nunes et al., 2017; Ribeiro et al., 2018).

The risk associated with the activity and vulnerability of FSWs determines the dynamics of HIV-1 and HTLV-1/2 dissemination. Prevention and control measures, including screening, diagnosis and treatment of STI and diseases, should be offered routinely as part of a comprehensive approach to decrease the impact these retroviruses among FSWs and other relevant key population groups (World Health Organization [WHO], 2016). The historical absence of routine actions will continue to facilitate the spread of HIV-1 and HTLV-1/2 among FSWs and in the general population in the state of Pará. Similarly, age ≥27 years associated with HIV-1 infection reflects that the longer the time spent as a sex worker, the greater the risk of acquiring the virus. The lack of analysis on the long duration of prostitution is one of the limitations of this study.”

The information obtained in a retrospective point of view is useful to clearly show the relevance of periodical surveillance using prevalence studies and molecular subtyping of HIV-1 and HTLV-1, which show the evolution of virus dissemination within a key population group in a large geographical region of Brazil that still carries the burden of limited infrastructure and access to health services. In conclusion, seroprevalences of HIV-1 and HTLV-1 among FSWs, were higher than what is commonly found in the general population, in the state of Pará, Brazil, with a higher occurrence in the state capital, Belem than in the neighboring cities. Age ≥27 years, unprotected sex and use of illicit drugs have been associated with viral infections. Subtypes B and Cosmopolitan have been identified in most FSWs infected with HIV-1 and HTLV-1, respectively. This information corroborates the epidemiological status of these viruses in Brazil and indicates the urgent need for actions to control, prevent and treat these infections among FSW in northern Brazil. Neglect of these facts may be causing the spread of the two viruses and their respective subtypes in the general population. This is a warning to public health authorities of the urgent call for specific actions to prevent and control these retroviruses among FSWs. Neglecting the health promotion of key populations is significantly contributing to the dissemination of these viruses in the north of Brazil.

## Data Availability Statement

The datasets presented in this study can be found in online repositories. The names of the repository/repositories and accession number(s) can be found in the article/Supplementary Material.

## Ethics Statement

The studies involving human participants were reviewed and approved by Ethics Committee on Human Research of the Fundação Centro de Hemoterapia e Hematologia do Pará (Protocol Number 01/2005). The patients/participants provided their written informed consent to participate in this study.

## Author Contributions

RLS, MOGI, and LFAM: conceptualization. MVSP, RMS, JBLS, DCLG, JCM, LQS, ALBL, and RSF: standard operating protocol development. MOGI, RI, and LFAM: supervision of the project. ABO-F and LFAM: statistical analysis. MOGI, RI, and LFAM: manuscript drafting. LFAM: project administration.

## Conflict of Interest

The authors declare that the research was conducted in the absence of any commercial or financial relationships that could be construed as a potential conflict of interest.

## Footnotes

1 http://tree.bio.ed.ac.uk/software/figtree
