# Supplementary material for: Molecular Epidemiology of HIV-1 and HTLV-1/2 Among Female Sex Workers in Four Cities in the State of Para, Northern Brazil
Source: Front Microbiol. 2020 Nov 11;11:602664. doi: 10.3389/fmicb.2020.602664 (PMC7686468; doi:10.3389/fmicb.2020.602664)
Supplement: Supplementary file 1 [file Table_1.DOCX]

Table S1. Bivariate and multivariate analysis of factors not associated with HIV among female sex workers in the state of Pará, northern Brazil.

| Characteristics | N | HIV + (%) | HIV - (%) | Bivariate Analysis | | Multivariate Analysis | |
| --- | --- | --- | --- | --- | --- | --- | --- |
|  |  |  |  | *p-value* | OR (95% CI) | *p-value* | OR (95% CI) |
| Total | 339 | 8 (2.4) | 331 (97.6) | - | - | - | - |
| Heterosexual | 264 | 8 (3.0) | 256 (97.0) | 0.8 | 2.0 (0.3 - 15.6) | 0.7 | 1.8 (0.4 - 11.4) |
| Up to 8 years of study^+^ | 282 | 8 (2.8) | 274 (97.2) | 0.9 | 1.4 (0.2 - 11.5) | 0.7 | 1.6 (0.3 - 9.8) |
| Single | 298 | 8 (2.7) | 290 (97.3) | 0.6 | 0.9 (0.1 - 7.9) | 0.8 | 1.2 (0.3 - 8.5) |
| Up to 1 wage per month | 145 | 4 (2.8) | 141 (97.2) | 0.9 | 1.3 (0.3 - 5.4) | 0.6 | 1.3 (0.3 - 5.4) |
| More than 10 clients per week | 94 | 5 (5.3) | 89 (94.7) | 0.1 | 4.5 (1.0 - 19.3) | 0.1 | 5.2 (0.9 - 17.7) |

OR: Odds ratio. 95% CI: 95% confidence intervals. ^+^ Including FSWs illiterate (no year of study).
