# Supplementary material for: Molecular Epidemiology of HIV-1 and HTLV-1/2 Among Female Sex Workers in Four Cities in the State of Para, Northern Brazil
Source: Front Microbiol. 2020 Nov 11;11:602664. doi: 10.3389/fmicb.2020.602664 (PMC7686468; doi:10.3389/fmicb.2020.602664)
Supplement: Supplementary file 2 [file Table_2.DOCX]

Table S2. Bivariate and multivariate analysis of factors not associated with HTLV among female sex workers in the state of Pará, northern Brazil.

| Characteristics | N | HTLV + (%) | HTLV - (%) | Bivariate Analysis | | Multivariate Analysis | |
| --- | --- | --- | --- | --- | --- | --- | --- |
|  |  |  |  | *p-value* | OR (95% CI) | *p-value* | OR (95% CI) |
| Total | 339 | 6 (1.8) | 333 (98.2) | - | - | - | - |
| Age ≥ 27 years | 59 | 3 (5.1) | 56 (94.9) | 0.1 | 4.9 (0.9 - 21.4) | 0.2 | 3.2 (0.8 - 28.5) |
| Heterosexual | 264 | 6 (2.3) | 258 (97.7) | 0.9 | 1.6 (0.3 - 13.5) | 0.8 | 1.8 (0.4 - 12.4) |
| Up to 8 years of study^+^ | 282 | 5 (1.8) | 277 (98.2) | 0.6 | 0.9 (0.1 - 8.7) | 0.4 | 0.9 (0.3 - 8.1) |
| Single | 298 | 5 (1.7) | 293 (98.3) | 0.8 | 0.7 (0.1 - 6.9) | 0.8 | 0.8 (0.4 - 7.3) |
| Up to 1 wage per month | 145 | 2 (1.4) | 143 (98.6) | 0.9 | 0.7 (0.1 - 3.6) | 0.9 | 0.7 (0.2 - 4.0) |
| More than 10 clients per week | 94 | 1 (1.1) | 93 (98.9) | 0.9 | 0.5 (0.1 - 4.5) | 0.4 | 0.7 (0.2 - 4.7) |

OR: Odds ratio. 95% CI: 95% confidence intervals. ^+^ Including FSWs illiterate (no year of study).
